# Toxicological evaluation of therapeutic and supra-therapeutic doses of Cellgevity® on reproductive function and biochemical indices in Wistar rats

**DOI:** 10.1186/s40360-018-0253-y

**Published:** 2018-10-25

**Authors:** O. Awodele, W. A. Badru, A. A. Busari, O. E. Kale, T. B. Ajayi, R. O. Udeh, P. M. Emeka

**Affiliations:** 1 Toxicology Unit, Department of Pharmacology, Therapeutics and Toxicology, College of Medicine, University of Lagos, PMB 12003, Idi-Araba Campus, Lagos, Nigeria; 2 Department of Pharmaceutical Sciences, College of Pharmacy, King Faisal University Hofuf, Hofuf, Kingdom of Saudi Arabia

**Keywords:** Cellgevity, Dietary supplements, Therapeutic dose, Supra-therapeutic dose, Toxicological profile, Subchronic toxicity study

## Abstract

**Background:**

The misconception about dietary supplements being safe has led many into the in-patient wards. Cellgevity® (CGV) is a Max International premiere antioxidant supplement formula used by a large population. This study evaluated the effects of therapeutic and supra-therapeutic doses of CGV on reproductive function and biochemical indices in Wistar rats.

**Methods:**

Seventy-two Wistar rats weighing 130 ± 15.8 g were grouped into two categories (male or female) of six rats per group. Control group received distilled water (10 ml/kg). Others received therapeutic (14.3 mg/kg or 28.6 mg/kg) and supra-therapeutic CGV doses (1000, 2000 or 3000 mg/kg) body weight per oral respectively.

**Results:**

After 60 days, supra-therapeutic doses of CGV reduced sperm motility (*p* < 0.05) by 31.8%, 31.3% and 34.5% respectively and increased (*p* < 0.05) abnormality in sperms by 200%, 241% and 141.3% respectively. CGV altered male (luteinizing, follicle stimulating hormones and testosterone) and female reproductive hormones (luteinizing, follicle stimulating hormones estrogen and progesterone) respectively. Therapeutic doses of CGV elevated reduced glutathione, superoxide dismutase, catalase and glutathione S-transferase, although, this was exceeded by supra-therapeutic doses and more in females than male rats. Supra-therapeutic dose (3000 mg/kg CGV) decreased body weight in both male and female rats by 50% (F(1.5, 30) = 1.2, *p* = 0.041) and 62.7% (F(2.1, 30) = 0.38, *p* = 0.038) respectively in treated rats. Supratherapeutic (3000 mg/kg) dose of CGV increased (*p* < 0.05) creatinine level by 99.1% while serum total protein was reduced (*p* < 0.05) by 60.1% (2000 mg/kg) and 57.2% (3000 mg/kg) respectively in male animals. In Female rats, supra-therapeutic doses of CGV elevated creatinine levels by 72.2% (1000 mg/kg), 60.2% (2000 mg/kg) and 124.8% (3000 mg/kg) respectively and 3000 mg/kg produces elevated serum low density lipoprotein by 34.6% in treated rats. Serum cholesterol, triglycerides, albumin, alkaline phosphatase were unaltered by CGV dosing. Histology shows seminiferous tubules with reduced spermatogenic cells. Also, female rat kidney revealed acute tubular necrosis at highest dose used in this study.

**Conclusion:**

Overall, these data suggest that pro-oxidant potential of the supra-therapeutic CGV doses is evident. Hence, it is necessary that its administration be done with caution using appropriate doses.

## Background

A complementary and alternative and medicine (CAM) practice has been promoting dietary supplements as one of the most easily accessible adjunct therapies acceptable worldwide [1]. This is in commonplace with a general theme underlying a majority of alternative therapies in their emphasis on natural modes of healing. However, certain problems have been reported concerning the consumption of these supplements, which include inappropriate use, excessive intake, and non-disclosure of amount being present in dietary supplements being used by patients resulting in concomitant use of various oversaturated dietary supplements with or without orthodox counterparts [2]. Cellgevity® (CGV) is one of the most widely used glutathione supplement that have been considered to be harmless, nevertheless, this general assumption should not be overlooked. It is marketed so as to salvage for the body glutathione and/or complements its production [3]. RiboCeine which is the active compound in CGV comprises mainly of D-Ribose and L-Cysteine. However, other components that are of scientific interest are; vitamin c, selenium, alpha lipoic, broccolli seed extract, curcumin, resveratrol, grape seed extract, quercetin, milk thistle seed extract, cordyceps, black pepper, aloe leaf. The manufacturer acclaimed ultimate reduced glutathione (GSH) enhancing effects for its use and various antioxidant supplementation studies have revealed a beneficial trend [4, 5]. This is because GSH is an important antioxidant that plays a major role in the biotransformation process in the body [6]. Evidence abounds that levels of GSH in the body decreases naturally with age [7]. Both stressful life style and some artificial components of foods are the popular culprits [8]. Due to some theoretical benefits of maintaining antioxidant defenses status, several approaches to increase systemic and/or tissue-specific glutathione concentrations have been of scientific interests. We now know that thiol-disulfides redox balancing is a major work of GSH in various body cells [9]. However, reduction of reactive oxygen or even nitrogen species by sulfhydryls enhances transcriptional activation, whereas oxidation inhibits activation [10]. Another important role of GSH supplementations are abilities to promote hypoxic apoptosis and improve protein function in a perturbed environment thereby yielding survival pathway [11]. In respect, the diversify roles of GSH supplementations have been well discussed [12]. Denovo alteration in antioxidant levels has been implicated in various diseases including those affecting the central nervous system [13]. The safety of some dietary supplements, such as protein supplements and others, remain a safety concern. The body’s ability to absorb nutrients is known to decrease with age and exposure to toxins takes its toll [14]. The aforementioned play a role in decreasing the Glutathione levels unless steps are taken to offset this process. Previous studies have reported that dietary supplements are now being used to prevent and treat various diseases [15, 16]. However, a number of adverse events have been reported over the past few years with the use of herbs and herbal products [17, 18]. Furthermore, concerns over drug-herbs interactions and irrational drug use (high dose) of CAM have arisen. In this present study, we investigated the toxicological activities of CGV using therapeutic and supra-therapeutic doses following a subchronic administration in Wistar rats.

## Methods

### Drugs and chemicals

Cellgevity (Riboceine®) was purchased in bulk from a subsidiary of Max international, Ikeja, Lagos state and it was the sole agent administered in this present study. Thiobarbituric acid (TBA), Ellman’s reagent (DTNB) and 1-Chloro-2,4,-dinitrobenzene (CDBN) from Sigma (USA) were purchased from Sigma Chemical Company (USA). Reduced glutathione (GSH), Metaphosphoric acid and Trichloroacetic acid (TCA) were purchased from J.I. Baker (USA). Bovine serum albumin fraction V (BSA) was purchased from SRL, India. Rat Follicle Stimulating Hormone (FSH) (Cat. No.: Rshakrfs-010R) and Luteinizing Hormone (LH) ELISA (Rshakrlh-010SR) kits were purchased from (Biovendor, Shibayagi Co., Ltd. (Japan). RAT Testosterone (RTC001R) ELISA was obtained from Biovendor, Laboratorni, medicinaa.sKarasek (Czech Republic). Sodium hydroxide was obtained from MERCK (Germany). All other chemicals and reagents used were of analytical grades. Atomic UV/ Visible Spectrophotometer obtained from JENWAY, Bibby Scientific (Model 7300 and 7305) (USA).

### Animals

Adult Wistar rats weighing between 100g and 153 g used in this study were purchased and nursed in the Laboratory Animal Centre of the College of Medicine, University of Lagos, Nigeria. The rats were housed under controlled conditions in the experimental animal handling facility of the College of Medicine, University of Lagos, Nigeria. The experimental animal room had a 12 h light/12 h dark schedule and maintained at a temperature of 22 ± 3 °C throughout the study. Animals were fed with commercially available rat pelleted diet (Livestock Feed Plc., Lagos, Nigeria) and were allowed access to water ad libitum throughout the period of the experiment. The experimental protocols were approved by the Institutional Animal Care and Use Committee, Department of Pharmacology, Therapeutic and Toxicology, College of Medicine, University of Lagos. Animals were certified fit for the experiment by the Institution’s Animal Health Officers before the commencement of the study. Beddings were changed on alternate days and the animals were sacrificed in a humane manner at the end of the experiment by cervical dislocation. The investigation conforms to the Guide for the Care and Use of Laboratory Animals published by the U. S. National Institutes of Health (NIH Publication No. 85–23, revised 1996)” for studies involving experimental animals and the procedures as documented by Kilkenny et al. [19] for reporting animal research.

### Experimental design and necropsy

Seventy-two (72) Wistar rats of both sexes  weighing 130 ± 15.8 g were grouped into two categories (male or female) of six (6) rats per group. Controls groups received distilled water (10 ml/kg). Other groups received therapeutic (14.3 mg/kg or 28.6 mg/kg) and supratherapeutic doses (1000, 2000 or 3000 mg/kg) body weight per oral respectively. Treatments lasted for 60 days. Rats were weighed weekly throughout the course of the experiment. Twenty four hours after the last administration, blood samples were obtained by ocular puncture into either lithium heparin or ethylene diaminetetraacetic acid (EDTA) bottles and animals were subsequently sacrificed by cervical dislocation. The anticoagulated blood samples were centrifuged at 4200 rpm for 5 min to separate the plasma from which all biochemical assays were carried out. The testis and epididymis were all harvested, weighed and homogenized in four volumes of buffer solution (0.1 M, pH 7.4). A portion of each organ was taken out for histology. The remaining was weighed and homogenized for biochemical assays.

### Analysis of sperm characteristics and morphology

The testes from each rat were carefully exposed and removed along with its adjoining epididymis. The slides on which the sperm cells were counted were heated to 37 °C until the time of the analysis. The analysis was carried out at room temperature using one epididymis of each rat. The left testis was separated from the epididymis and the caudal epididymal tissue was removed and placed in a petri dish containing 1 mL normal saline solution. An incision of about 1 mm was made in the caudal epididymis to liberate its spermatozoa into the saline solution. Progressive sperm motility, sperm count, and sperm viability were then examined under the microscope attached to a Celestron® Digital Microscope Imager (Torrance, CA 90503) and viewed under X40 objective as described elsewhere [18]. Epididymal sperm motility was assessed by calculating motile spermatozoa per unit area and was expressed as percentage motility. Epididymal sperm count was done using the improved Neubauerhemocytometer and expressed as million/ml of suspension. The sperm viability was also determined using Eosin/Nigrosin stain. The motile (live) sperm cells were unstained while the non-motile (dead) sperms absorbed the stain. The stained and unstained sperm cells were counted and an average value for each was recorded from which percentage viability was calculated. Sperm morphology was evaluated by staining the sperm smears on microscope slides with two drops of Walls and Ewa stain after they were air-dried. The slides were examined under the microscope under oil immersion with X 100 objectives.

#### Assessments of oxidant/antioxidant status

Lipid peroxidation activity was determined by measuring the formation of thiobarbituric acid reactive substances according to the method of Varshney and Kale [20] The method of Beutler et al. [21] was used for the determination of activity of reduced glutathione. While glutathione S-transferase activity was determined according to the method described by Habig et al. [22]. The levels of superoxide dismutase and catalase activities were determined by the method of Misra and Fridovich [23] and Sinha [24] respectively.

### Reproductive hormone assessments

Serum concentrations of male reproductive hormones were measured using micro plate enzyme-linked immunosorbent assay (ELISA) and expressed as Units/l. Studies protocols were discussed below.

### Rat FSH and LH ELISA

Briefly, in Rat FSH or LH ELISA Kit, biotin-conjugated anti- FSH/anti-LH and standard or sample were incubated in monoclonal anti-FSH antibody-coated wells. After 15 h incubation and wash-ing, HRP (horse radish peroxidase)-conjugated avidin was added, and incubated for 30 min. After washing, HRP-complex remaining in wells was reacted with a chromogenic substrate (TMB) for 20 min, and reaction was stopped by addition of acidic solution, and absorbance of yellow product was measured spectrophotometrically at 450 nm. Triplicates samples of LH/FSH were tested twice on one plate, respectively (Intra-Assay: CV < 8% and inter-assay: CV < 10%). The absorbance is nearly proportional to FSH or LH concentration. FSH or LH concentrations in unknown samples were then extrapolated via given their respected standard curve [25].

### Rat testosterone ELISA

A 10 μl of each of sample with new disposable tips into appropriate wells was dispensed in a 100 μl of incubation Buffer into each well. Added was a 50 μl enzyme Conjugate into each well which was incubated for 60 min at room temperature on a microplate mixer. This was discarded and the well rinsed 4 times with diluted washing solution (300 μl per well). Then 200 μl was added of substrate solution to each well and incubated standing for 30 min in the dark. Triplicates samples of testosterone was tested twice on one plate, respectively (Intra-Assay: CV < 15% and inter-assay: CV < 15%). The reaction was stopped by adding 50 μl of stop solution to each well and the absorbance determined for each well at 450 nm [26].

### Rat estrogen ELISA

The blank well and or 50 μl of standard in triplicate or sample per well were prepared. Then added was a 50 μl of HRP-conjugate to each well except for the blank. Then 50 μl antibody was added to each well. The solution was thorough mixed, and then incubated for 3 h at 37 °C. Each well was washed with buffer (350 μl), wait 10 s and spin. Following the last wash, remaining wash buffer was aspirated. Plate was inverted and blotted against clean paper towels. A 50 μl of substrate A was added and substrate B to each well, and mixed. Solution was incubated for 15 min at 37 °C. Triplicates samples of estrogen was tested twice on one plate, respectively (Intra-Assay: CV < 8% and inter-assay: CV < 12%). In a dark environment, a 50 μl of stop solution was added to each well. The color change appeared uniform. Determination of the optical den-sity was carried out within 10 min, using a microplate reader set to 450 nm [27].

### Rat progesterone ELISA

Rat progesterone ELISA A dispensed 25 μl of each sample with new disposable tips into appropriate wells and added 50 μl of incubation Buffer into each well. Added was a 100 μl enzyme conjugate into each well and incubated for 1 h at 22 ± 2 °C on a microplate mixer. Following a thorough rinsing up to 4 times with diluted wash solution, a 200 μl of substrate solution was added to each well and incubated for 30 min in the dark. Triplicates samples of progesterone was tested twice on one plate, respectively (Intra-Assay: CV < 10% and inter-assay: CV < 10%). This reaction was terminated by the addition of a 50 μl of stop solution to each well. The progesterone level was measured at 450 nm within 15 min [27].

#### Assessment of biochemical parameters

Plasma alkaline phosphate was carried out according to the method described by Roy [28] to assess liver function. Renal function was assessed by measuring plasma creatinine levels and blood urea nitrogen was assayed following the method of Fossati et al. [29] and Skegg’s [30]. Total serum protein concentration was determined according to the principles based on the Biuret reaction. Albumin concentrations were determined according to the principles based on the bromocresol green reaction [31] respectively. Uric acid levels were determined according to the methods of Fossati et al. [32]. Total plasma cholesterol and Triglyceride concentrations were estimated following the method described by Trinder [33] by using commercial kits obtained from Randox Laboratories Ltd. (Crumlin, UK). High-Density Lipoprotein was estimated according to Warnick and Albers [34] while serum low-density lipoprotein was calculated using Freidewald formula [35].

### Statistics

Results were expressed as mean ± standard error of mean (SEM). Differences between groups were determined by one-way analysis of variance (ANOVA) using Statistical Package for Social Sciences (SPSS, 20.0) software for windows. Post hoc testing was performed for intergroup comparisons using the least significant difference (LSD), followed by Dunnett’s test, and the two-tailed *p*-value < 0.05 was considered significant. Figures were obtained using GraphPad Prism 6.

## Results

### Acute toxicity test

CGV acute toxicity study followed the guidelines according to the OECD Test Guidelines on Acute Oral Toxicity No 420 (OECD, 1992) [36]. An acute intragastric administration of 500, 1000 and 2000 mg/kg doses body weight (*n* = 5 per sex) of CGV in mice did not show physically observed adverse reactions on the fur, skin, subcutaneous swelling, eyes dullness, eyes opacities, colour and consistency of feaces, teeth and breathing abnormalities. However, mice administered 4000 mg/kg of CGV orally demonstrated reduced locomotion, dullness, raised of fur, and tears respectively. In addition, the group administered 4 g/kg had 20% mortality. Thus, 1000, 2000, and 3000 mg/kg were used for supra-therapeutic administration.

**Fig. 1:**
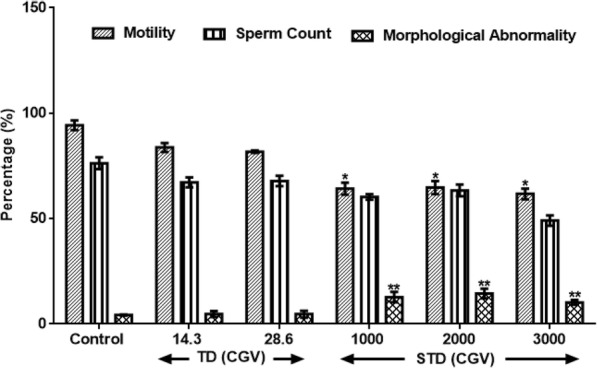
Effect of therapeutic and supra-therapeutic doses of CGV on Sperm Motility, Sperm Count and Sperm Morphology Abnormality) in male rats. Data are expressed as mean ± SEM. *n* = 6. CGV: Cellgevity®, TD: Therapeutic dose; STD: Supra-therapeutic dose. ^*^p < 0.05 or ^**^p < 0.001 when compared with control group. Control (DW: Distilled Water, 10 ml/kg)

Figure 1 results show the effects of therapeutic and supra-therapeutic doses of Cellgevity (Ribceine) on sperm motility, sperm count and morphology abnormality in male rats. Therapeutic doses of CGV neither alter sperm motility nor counts when compared with control distilled water group. In contrast, supra-therapeutic doses of 1000 mg/kg, 2000 mg/kg and 3000 mg/kg reduced sperm motility (F(5.2, 30) = 1.5, *p* < 0.048) by 31.8%, 31.3% and 34.5% respectively. Also, 3000 mg/kg CGV reduced sperm counts by 3.7%. CGV administration at 1000 mg/kg, 2000 mg/kg and 3000 mg/kg produce increased (F(1.4, 28) = 0.48, *p* = 0.001–0.002) abnormality in sperms by 200%, 241% and 141.3% respectively.

**Fig. 2:**
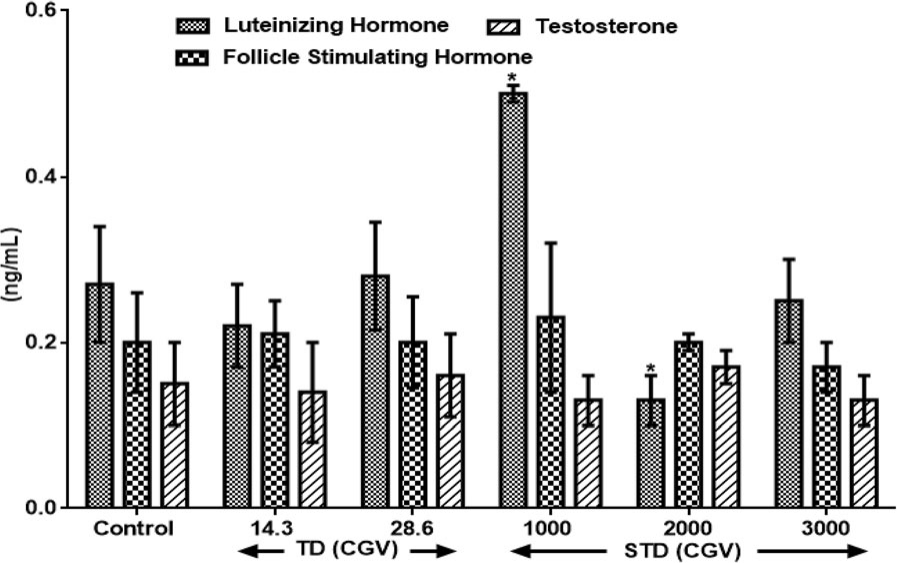
Effects of therapeutic and supra-therapeutic doses of CGV on male reproductive hormones in rat. Data are expressed as mean ± SEM. *n* = 6. CGV: Cellgevity®, TD: Therapeutic dose; STD: Supra-therapeutic dose. ^*^p < 0.05 when compared with control group. Control (Distilled Water, 10 ml/kg); Follicle Stimulating Hormone; Luteinizing Hormone; Testosterone. Nanograms per decilitre (ng/dL)

Results in Fig. 2 show the effects of therapeutic and supra-therapeutic doses of CGV on male reproductive hormones in rat. CGV showed increased (F(4, 26) = 0.56, *p* = 0.034) LH at 1000 mg/kg while it shows decreased (F(1.3, 24) = 0.33, *p* < 0.151) at higher doses of 2000 and 3000 mg/kg by 51.85% and 7.4% respectively when compared with control. Similarly, FSH was increased at 1000 mg/kg by 15% (F(2, 28) = 2.6, *p* = 0.135). Both 2000 mg/kg and 3000 mg/kg of CGV did not alter FSH levels. Also, all the supra-therapeutic doses of CGV used in this study did not significantly alter testosterone levels when compared with control.

**Fig. 3:**
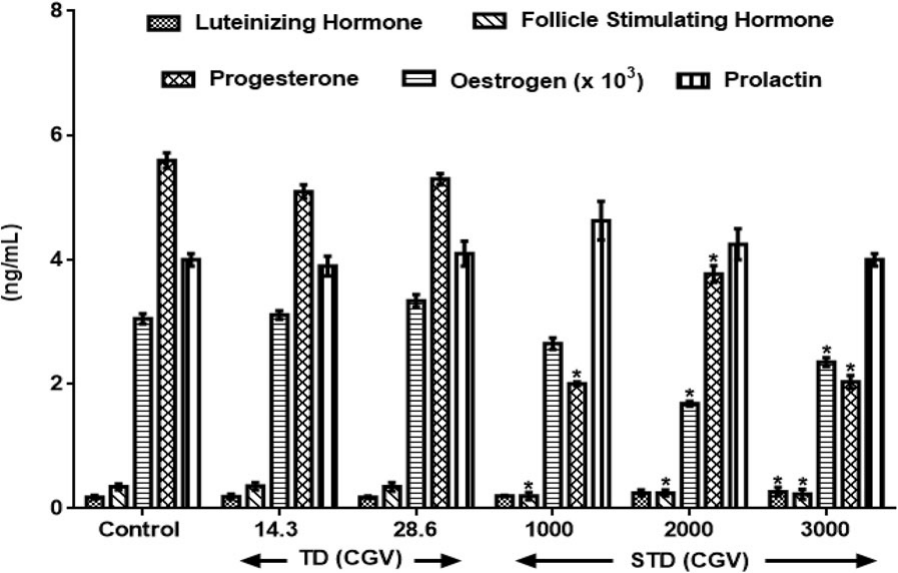
Effects of therapeutic and supra-therapeutic doses of CGV on female reproductive hormones in rat. Data are expressed as mean ± SEM. *n* = 6. CGV: Cellgevity®, TD: Therapeutic dose; STD: Supra-therapeutic dose. ^*^p < 0.05 when compared with control group. Control (Distilled Water, 10 ml/kg). Nanograms per decilitre (ng/dL)

Figure 3 results show the effects of therapeutic and supra-therapeutic doses of CGV on female reproductive hormones in rat. An administration of 1000 mg/kg and 2000 mg/kg slightly although insignificantly elevated (F(3.1, 25) = 31.6, *p* < 0.235) LH levels when compared with control, whereas, 3000 mg/kg increased (F (4, 24) = 0.37, *p* = 0.038) LH levels by 50%. Also, CGV doses of 1000 mg/kg and 3000 mg/kg decreased (*p* < 0.05) FSH levels by 42.9% and 34.3% respectively when administrated to rats. More so, a dose dependent decrease was obtained in PROG levels following the administration of 1000 mg/kg (F(5.3, 28) = 6.8, *p* < 13.1%), 2000 mg/kg (F(2.2, 27) = 0.88, *p* = 0.042; 44.8%) and 3000 mg/kg (*p* < 0.05, 64%) respectively in treated animals. Similar decrease was observed with ESTRL at 1000, 2000 and 3000 mg/kg by 64.3%, 32.7% and 64% respectively. However, there were no changes observed in the PRL level in all rats.

Results in Table 1 show the effects of therapeutic and supra-therapeutic doses of CGV on oxidative stress parameters and antioxidant indices in rat testis. Lipid peroxidation, a biomarker of oxidative stress, measured as malondialdehyde (MDA) was unaltered given the therapeutic doses of CGV (14.3 and 28.6 mg/kg) when compared with control distilled water group as observed in this study. Similarly, given the supra-therapeutic doses of CGV, there was no significant alteration in MDA levels. Both doses of CGV, 14.3 mg/kg and 28.6 mg/kg, increased (F(3, 30) = 1.6, *p* < 0.061) GSH (14.5% and 107%), SOD (121.6% and 54.5%), CAT (85.1% and 107.7%) and GST (44.1% and 16.9%) respectively when compared with control distilled water group. CGV at dose of 2000 mg/kg reduced GST activity by 35.1%. Furthermore, given 3000 mg/kg CGV, there was significantly reduced (F(4, 22) = 0.38, *p* < 0.05) SOD (34.7%) and GST (44.4%) activities respectively.

**Table 1:** Effects of therapeutic and supra-therapeutic doses of CGV on antioxidant indices in rat testes

Treatment	GSH	SOD	CAT	GST	MDA
Control (DW, 10 ml/kg)	21.27 ± 0.98	1.67 ± 0.14	13.00 ± 0.95	5.4 ± 0.03	1.37 ± 0.02
14.3 mg/kg	24.35 ± 1.98 (− 14.5)	3.70 ± 0.16^*^ (− 121.6)	24.06 ± 1.98^*^(− 85.1)	7.78 ± 0.52^*^(44.1)	1.12 ± 0.03 (18.2)
28.6 mg/kg	44.05 ± 1.24^**^(107.1)	2.58 ± 0.34^*^(− 54.5)	27.00 ± 1.04^*^(− 107.7)	6.31 ± 0.04(− 16.9)	1.17 ± 0.01 (14.6)
1000 mg/kg	24.58 ± 1.25 (− 15.6)	1.61 ± 0.33 (3.6)	14.81 ± 0.44 (− 13.9)	4.40 ± 0.09 (18.5)	1.36 ± 0.02 (0.7)
2000 mg/kg	20.48 ± 0.55 (3.7)	1.31 ± 0.02 (21.6)	14.51 ± 0.93(− 11.6)	3.50 ± 0.01^*^(35.1)	1.45 ± 0.01 (− 5.8)
3000 mg/kg	19.33 ± 0.68 (10)	1.09 ± 0.15* (34.7)	10.78 ± 0.95 (17.1)	3.00 ± 0.04^*^ (44.4)	1.35 ± 0.05 (1.5)

Data are expressed as mean ± SEM. *n* = 6. ^*^p < 0.05 or ^**^p < 0.001 when compared with control group. Values in parenthesis represent % change; (−) increase; (+) decrease. Control (Distilled Water, 10 ml/kg); Superoxide dismutase; *GSH* Reduced glutathione (μmol/mg protein), *SOD* Superoxide dismutase (μmol/min/mg protein), Catalase (μmol/ml/mg protein), *GST* Glutathione S-transferase (μmol/ml/mg protein), *MDA* Malondialdehyde (nmol/mg protein)

Results in Table 2 show the effects of therapeutic and supra-therapeutic doses of CGV on oxidative stress parameters and antioxidant indices in rat ovaries. Lipid peroxidation, a biomarker of oxidative stress, measured as malondialdehyde (MDA) was unaltered given the therapeutic doses of CGV (14.3 and 28.6 mg/kg) as observed in this study. However, at supra-therapeutic doses of 1000 mg/kg, 2000 mg/kg and 3000 mg/kg of CGV, an increased MDA of 32.7%, 28.3% and 38.9% were obtained respectively when compared with control animals. Interestingly, therapeutic but not supra-therapeutic doses of CGV improved GSH significantly (F(5.5, 27) = 0.74, *p* < 0.003) by 172% and 181.3% respectively. Also, catalase levels given the 14.3 mg/kg, 28.6 mg/kg, 1000 mg/kg, 2000 mg/kg and 3000 mg/kg doses of CGV increased (F(3.4, 30) = 13.6, *p* < 0.315) by 32.2%, 35.4%, 3.5%, 0.6% and 7.5% respectively in the treated rats. In contrast, SOD and GST activities were insignificantly decreased (*p* > 0.05) both at the therapeutic as well as supra-therapeutic doses of CGV in rats.

**Table 2:** Effects of therapeutic and supra-therapeutic doses of CGV on antioxidant indices in rat ovaries

Treatment	GSH	SOD	CAT	GST	MDA
Control	21.94 ± 1.77	17.9 ± 0.13	12.47 ± 1.46	6.3 ± 0.05	1.13 ± 0.02
14.3	59.68 ± 3.77^**^ (− 172)	13.21 ± 1.06 (26.2)	16.48 ± 2.58^*^ (− 32.2)	3.95 ± 1.15 (37.3)	1.13 ± 0.22 (0.0)
28.6	61.72 ± 5.19^**^ (− 181.3)	13.78 ± 2.17 (23)	16.89 ± 1.04^*^ (− 35.4)	3.98 ± 1.49 (36.8)	0.93 ± 0.13 (17.7)
1000	18.31 ± 1.00 (16.5)	13.9 ± 0.12 (22.3)	12.91 ± 0.67 (− 3.5)	3.7 ± 0.03 (41.3)	1.5 ± 0.04^*^ (− 32.7)
2000	21.62 ± 1.96 (1.5)	12.8 ± 0.12 (28.5)	12.54 ± 0.41 (− 0.6)	3.4 ± 0.03 (46.0)	1.45 ± 0.04^*^(− 28.3)
3000	20.37 ± 1.51 (7.2)	16.0 ± 0.04 (10.6)	13.41 ± 0.47 (− 7.5)	4.2 ± 0.01 (33.3)	1.57 ± 0.02^*^(− 38.9)

Data are expressed as mean ± SEM. *n* = 6.^*^p < 0.05 or ^**^p < 0.001 when compared with control group. Values in parenthesis represent % change; (−) increase; (+) decrease. Control (Distilled Water, 10 ml/kg); Superoxide dismutase; *GSH* Reduced glutathione (μmol/mg protein), *SOD* Superoxide dismutase (μmol/min/mg protein), Catalase (μmol/ml/mg protein), *GST* Glutathione S-transferase (μmol/ml/mg protein), *MDA* Malondialdehyde (nmol/mg protein)

**Fig. 4:**
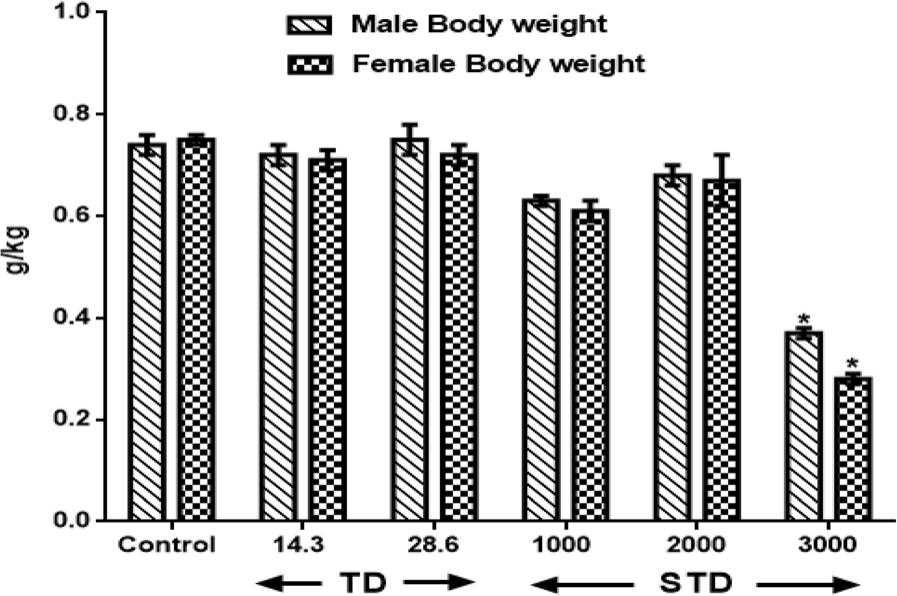
Effect of therapeutic and supra-therapeutic doses of CGV on Body Weight of male and female rats after a subchronic administration for 60 days. Data are expressed as mean ± SEM. *n* = 6. CGV: Cellgevity®, TD: Therapeutic dose; STD: Supra-therapeutic dose. ^*^p < 0.05 or ^**^p < 0.001 when compared with control group. Control (Distilled Water, 10 ml/kg)

Results in Fig. 4 show the difference in body weight following therapeutic and supra-therapeutic CGV administration in male and female rats. The initial weight of the animals and final weight were compared and expressed as g/kg body weight. An administration of CGV doses of 1000 and 2000 mg/kg produce insignificant decreased (F(2.6, 30) = 13.1, *p* < 0.217) body weight in rats of both sexes by 14.9%, 7.7% (male) and 23%, 10.7% (female) when compared with control distilled water group. However, at the maximum supra-therapeutic dose (3000 mg/kg) used in this study, there was decrease (F(4.1, 28) = 0.47, *p* < 0.05) in male and female rats by 50% and 62.7% respectively in treated rats.

The effects of therapeutic and supra-therapeutic doses of CGV on serum biochemical indices in male rats were assessed (Table 3). Therapeutic doses of CGV did not cause any change in urea and creatinine levels. However, 3000 mg/kg dose of CGV increased (F(5, 24) = 0.06, *p* = 0.0036) creatinine level by 99.1% when compared with control distilled water group. Also, neither serum total cholesterol nor triglycerides was altered following therapeutic and supra-therapeutic doses of CGV in treated rats. Both serum high density lipoprotein and low density lipoprotein remained unchanged in all animals. Alkaline aminotransferase level of treated rats was not significantly different from those of control distilled water group. In addition, serum total protein but not albumin levels was reduced (F(5.6, 26) = 0.89, *p* < 0.041) by 60.1% (2000 mg/kg) and 57.2% (3000 mg/kg) respectively.

**Table 3:** Effects of therapeutic and supra-therapeutic doses of CGV On Serum Biochemical Indices in Male Rats

Treatment/ mg/kg	UREA (mg/l)	CREAT (mmol/l)	CHOL (mmol/l)	TG (mol/l)	HDL (mmol/l)	LDL (mmol/l)	ALP (U/l)	ALB (U/l)	TP (U/l)
Control	10.55 ± 0.86	4.43 ± 0.76	1.64 ± 0.09	0.91 ± 0.16	0.57 ± 0.05	0.35 ± 0.04	18.90 ± 1.82	27.52 ± 1.81	13.71 ± 0.02
14.3	10.10 ± 0.21 (4.3)	4.11 ± 1.04 (7.2)	1.44 ± 0.22 (12.2)	0.89 ± 0.07 (2.1)	0.58 ± 0.02 (− 1.8)	0.33 ± 0.05 (5.7)	18.43 ± 2.02 (2.5)	26.25 ± 1.70 (4.6)	12.3 ± 0.47 (10.3)
28.6	10.90 ± 0.25 (3.3)	4.21 ± 1.03 (5.0)	1.38 ± 0.08 (15.9)	0.78 ± 0.11 (14.3)	0.52 ± 0.04 (8.8)	0.32 ± 0.03 (8.6)	18.06 ± 3.93 (4.4)	27.76 ± 2.05 (− 0.9)	11.47 ± 0.15 (16.3)
1000	9.58 ± 0.28 (9.2)	3.65 ± 0.99 (17.6)	1.57 ± 0.12 (4.3)	0.76 ± 0.07 (16.5)	0.55 ± 0.02 (3.5)	0.35 ± 0.02 (0)	18.32 ± 3.02 (3.1)	30.55 ± 3.70 (11)	13.3 ± 1.47 (3)
2000	9.50 ± 0.69 (10.0)	4.95 ± 2.13 (− 11.7)	1.57 ± 0.18 (4.3)	0.77 ± 0.11 (15.4)	0.59 ± 0.03 (− 3.5)	0.37 ± 0.07 (− 5.7)	17.66 ± 3.93 (6.6)	28.45 ± 3.05 (− 3.4)	5.47 ± 0.25* (60.1)
3000	9.90 ± 0.80 (10.1)	8.82 ± 1.75^*^ (− 99.1)	1.61 ± 0.21 (1.8 s)	0.75 ± 0.07 (17.5)	0.56 ± 0.03 (1.8)	0.4 ± 0.08 (− 14.3)	18.75 ± 1.8 (0.8)	28.50 ± 3.12 (− 3.6)	5.87 ± 1.11^*^ (57.2)

Data are expressed as mean ± SEM. *n* = 6. ^*^p < 0.05 or ^**^p < 0.001 when compared with control group. Values in parenthesis represent % change; (−) increase; (+) decrease. Control (Distilled Water, 10 ml/kg). *CREA* Creatinine, *CHOL* cholesterol, *TG* Triglycerides, *HDL* High density lipoprotein, *LDL* Low density lipoprotein, *ALP* Alkaline phosphatase, *ALB* Albumin, *TP* total protein

Results in Table 4 show the effects of therapeutic and supra-therapeutic doses of CGV on serum biochemical indices in Female Rats. The administration of CGV did not alter biochemical parameter levels of serum urea, total cholesterol, high density lipoprotein, alkaline phosphatase, albumin as well as total protein respectively. However, supra-therapeutic doses of CGV elevated (F(3.8, 28) = 0.12, *p* < 0.045) creatinine levels by 72.2% (1000 mg/kg), 60.2% (2000 mg/kg) and 124.8% (3000) respectively. Similarly, 3000 mg/kg of CGV increased serum low density lipoprotein by 34.6% in treated rats.

**Table 4:** Effects of therapeutic and supra-therapeutic doses of CGV On Serum Biochemical Parameters in Female Rats

Treatment/ mg/kg	UREA (mg/dl)	CREA (mmol/l)	CHOL (mmol/l)	TG (mol/l)	HDL (mmol/l)	LDL (mmol/l)	ALP (U/l)	ALB (U/l)	TP (U/l)
Control	9.47 ± 0.52	1.33 ± 0.40	1.31 ± 0.08	0.86 ± 0.06	0.58 ± 0.01	0.26 ± 0.04	16.18 ± 1.59	27.78 ± 1.60	9.72 ± 1.19
14.3	9.55 ± 0.42 (− 0.8)	1.29 ± 0.21 (3.0)	1.36 ± 0.09 (− 3.8)	0.87 ± 0.02 (− 1.1)	0.55 ± 0.02 (5.2)	0.27 ± 0.04 (− 3.8)	16.22 ± 1.78 (0.2)	31.03 ± 1.54 (− 12.7)	9.93 ± 1.01 (− 2.16)
28.6	9.16 ± 0.55 (−0.8)	1.13 ± 1.31 (15)	1.32 ± 0.02 (0.8)	0.77 ± 0.03 (10.5)	0.58 ± 0.03 (0)	0.28 ± 0.02 (− 7.7)	16.30 ± 1.29 (− 0.7)	30.44 ± 1.63 (− 9.6)	10.46 ± 1.22 (− 7.6)
1000	9.65 ± 0.32 (− 2.17)	2.29 ± 0.27^*^ (72.18)	1.46 ± 0.11 (− 11.5)	0.68 ± 0.03 (20.9)	0.56 ± 0.01 (3.4)	0.29 ± 0.03 (− 11.5)	16.22 ± 2.80 (− 0.2)	33.43 ± 2.54 (20)	10.32 ± 1.21 ()
2000	9.25 ± 0.78 (2.3)	2.13 ± 1.32^*^ (60.2)	1.32 ± 0.03 (− 0.8)	0.69 ± 0.04 (19.8)	0.59 ± 0.02 (− 1.7)	0.27 ± 0.03 (− 3.8)	16.30 ± 2.29 (− 0.7)	30.25 ± 3.63 (− 8.9)	11.36 ± 1.82 (− 6.2)
3000	9.90 ± 0.21 (− 4.5)	2.99 ± 2.07^*^ (− 124.8)	1.57 ± 0.09 (− 19.8)	0.72 ± 0.03 (16.3)	0.57 ± 0.01 (1.7)	0.35 ± 0.03* (34.6)	15.42 ± 1.44 (4.7)	29.98 ± 3.02 (− 7.9)	11.08 ± 1.25 (− 14.0)

Data are expressed as mean ± SEM. *n* = 6.^*^p < 0.05 or ^**^p < 0.001 when compared with control group. Values in parenthesis represent % change; (−) increase; (+) decrease. Control (Distilled Water, 10 ml/kg). *CREA* Creatinine, *CHOL* cholesterol, *TG* Triglycerides, *HDL* High density lipoprotein, *LDL* Low density lipoprotein, *ALP* Alkaline phosphatase, *ALB* Albumin, *TP* total protein

## Discussion

The demand for intake of antioxidant food or dietary antioxidant has increase in recent time with the hope to keep body healthy and free from diseases [37]. However, it is a great concern that people often take supplements containing antioxidants irrationally at higher doses than recommended. We reported the hypothesis that CGV which supposedly should provide a maximum antioxidant function by GSH synthesis rates and concentrations as expressed by the manufacturer could act as a pro-oxidant causing oxidative damage in normal humans particularly at the supra therapeutic doses following a subchronic administration in Wistar rats. Halliwell [38] describes antioxidant as any substance whose presence, even at low concentration may delays or inhibits the oxidation of a substrate. Antioxidant therapy exists long time ago and is still in the forefront of preventive medicine [39]. Taking into account the pro and cons of antioxidant enzymes on reproductive function and health, we cannot keep any longer our eyes closed on the potential adverse drug effects that could result from self-indulged overdose. CGV is a dietary supplementation of some key molecules, D-Ribose and L-Cysteine, which would increase intracellular GSH synthesis and concentrations and thus lower oxidative stress. Also, vitamin C, selenium, alpha lipoic, broccolli seed extract, curcumin, resveratrol, grape seed extract, quercetin, milk thistle seed extract, cordyceps, black pepper, aloe leaf are present to complement this effort [40]. Glutathione, the most abundant endogenous intracellular antioxidant, plays a central role in antioxidant defenses, and irreversible cell damage supervenes when the cell is unable to maintain intracellular glutathione concentrations [41]. However, reduction in intake of antioxidant substances may increase the chance of oxidative stress which may lead to cell damage [42]. Therefore, intake of such natural antioxidants may give protective effect against free radical induced diseases. Some reports have suggested that concentrations of glutathione decline with aging and in disease conditions [43, 44]. Also, the mechanisms of glutathione deficiency in relation to aging have been documented and have been suggested to be associated with an increased pro-oxidizing shift and elevated oxidative stress [45]. Still, how GSH could act as a pro-oxidant molecule is not well understood.

Vitamin C is vitally needed for cellular collagen [46] and neurotransmitters biosynthesis and plays important antioxidant defense against atherogenic, carcinogenic substance and neurodegeneration [47]. Selenium utilizes glutathione peroxidase to carry out an antioxidant function [48]. Thus, CGV which contains selenium could modulate the functional ability of selenoproteins. Broccoli contains much health promoting phytochemicals including glucosinolates with chemopreventive activity against cancer as well as having antioxidant properties [49]. More so, alpha lipoic acid is present naturally in edible meats, spinach, broccoli, potatoes, yams, carrots, beets, and even yeast [50]. It has a potent thiol-containing antioxidant and may contribute to the development of cardiovascular risk factors [51]. Alpha lipoic acid is actively involved in free radicals turnover, arrest inflammation and promote actions of endothelial relaxing factor in vesicles and endothelium [52]. Curcumin, resveratrol, grape seed extract, quercetin, milk thistle seed extract, cordyceps, black pepper, aloe leaf have been documented for antioxidants [53]. From the results obtained in our study, therapeutic doses of CGV neither alter sperm motility nor counts when compared with control group. However, in contrast, supra-therapeutic administration reduced sperm motility as well as sperm counts. Spermatozoa possess primarily enzymatic antioxidants, with superoxide dismutase being the most predominant [54]. Dietary antioxidants are usually present in the form of vitamins, carotenoids, and flavonoids. Metal-binding proteins such as albumin, ceruloplasmin, metallothionein, transferrin, ferritin, and myoglobin function by inactivating transition metal ions that otherwise would have catalyzed the production of free radicals [55]. In addition, CGV supra-therapeutic indication enhances abnormality of sperm cells and demonstrated such tendency to influence body hormones as observed in our results. CGV increases luteinizing and follicle stimulating hormones at the lowest supra-therapeutic dose employed in this study, although, this was reversed as the doses increases. This lack of effects due to higher doses of CGV could results from a saturation activity at lower doses, although, we did not assess the neuroendocrine effects of CGV. However, such feedback mechanisms could implicate some unidentifiable regions in the brain playing pro-antioxidant roles to supra-therapeutic dosing which may require further investigations. CGV showed increased LH at 1000 mg/kg while it shows decreased at higher doses of 2000 and 3000 mg/kg by 51.85% and 7.4% respectively when compared with control.

**Fig. 5:**
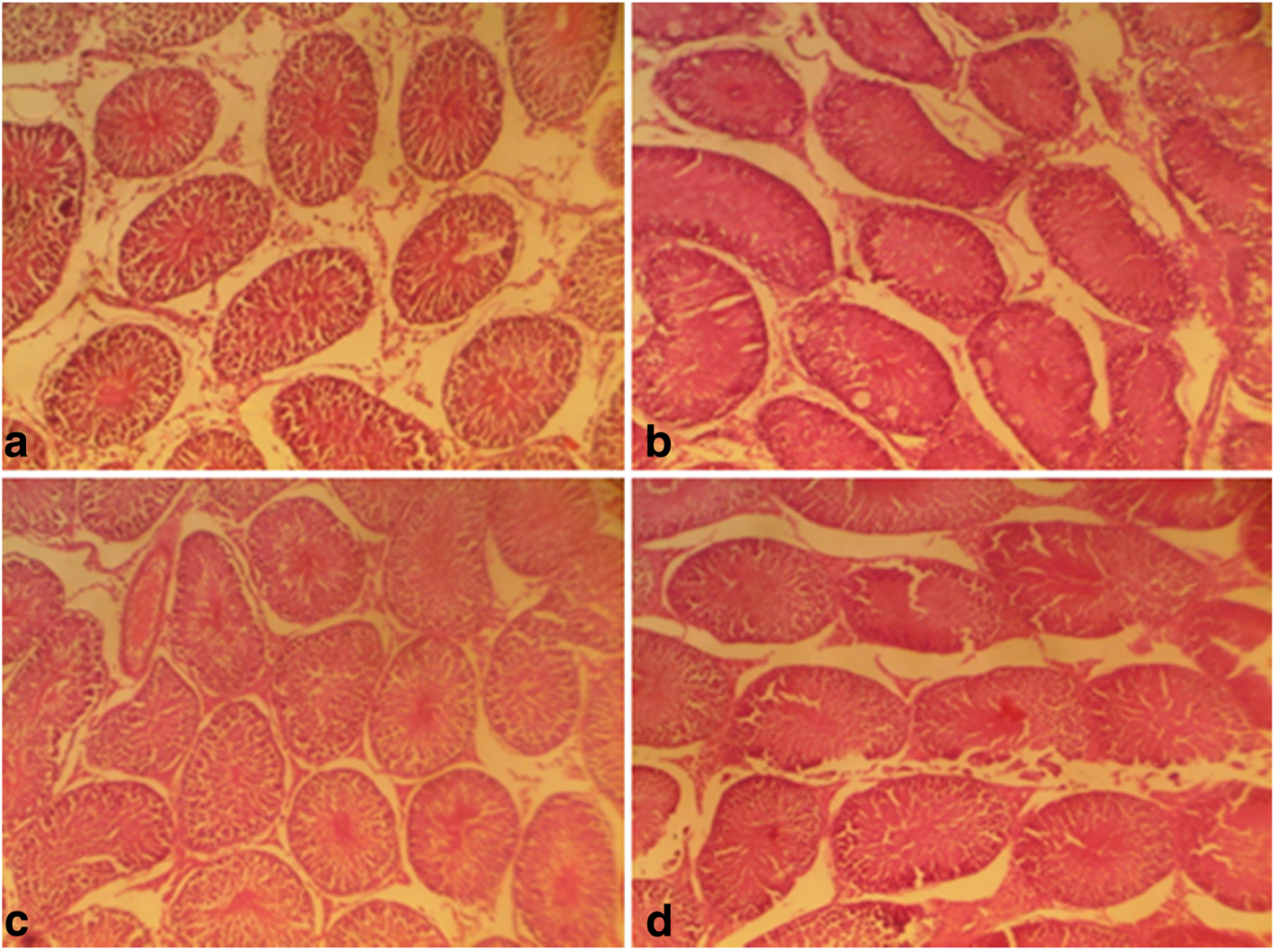
Male rat testes. Control shows normal testes (distilled water, 10 ml/kg) (**a**), 1000mg/kg CGV shows seminiferous tubules with reduced spermatogenic series (**b**), 2000mg/kg CGV seminiferous tubules with reduced spermatogenic series (**c**), 3000mg/kg CGV seminiferous tubules with reduced spermatogenic series (**d**). (H & E, × 400) (CGV: Cellgevity®)

**Fig. 6:**
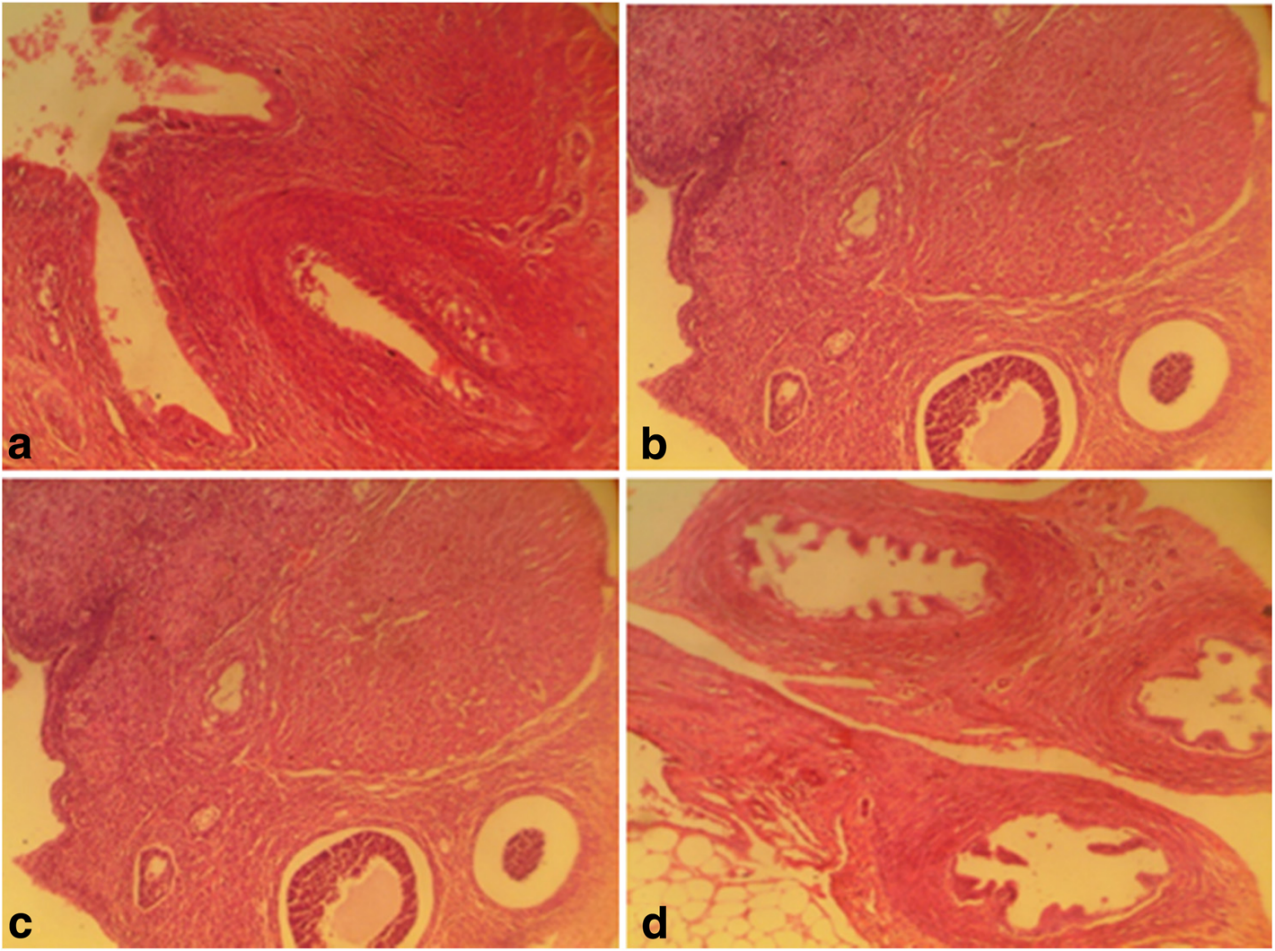
Female rat ovaries. Control shows normal ovaries (distilled water, 10 ml/kg) (**a**), 1000mg/kg CGV shows normal ovaries (**b**), 2000mg/kg CGV shows normal ovaries (**c**), 3000mg/kg CGV shows normal ovaries (**d**). (H & E, × 400) (CGV: Cellgevity®)

**Fig. 7:**
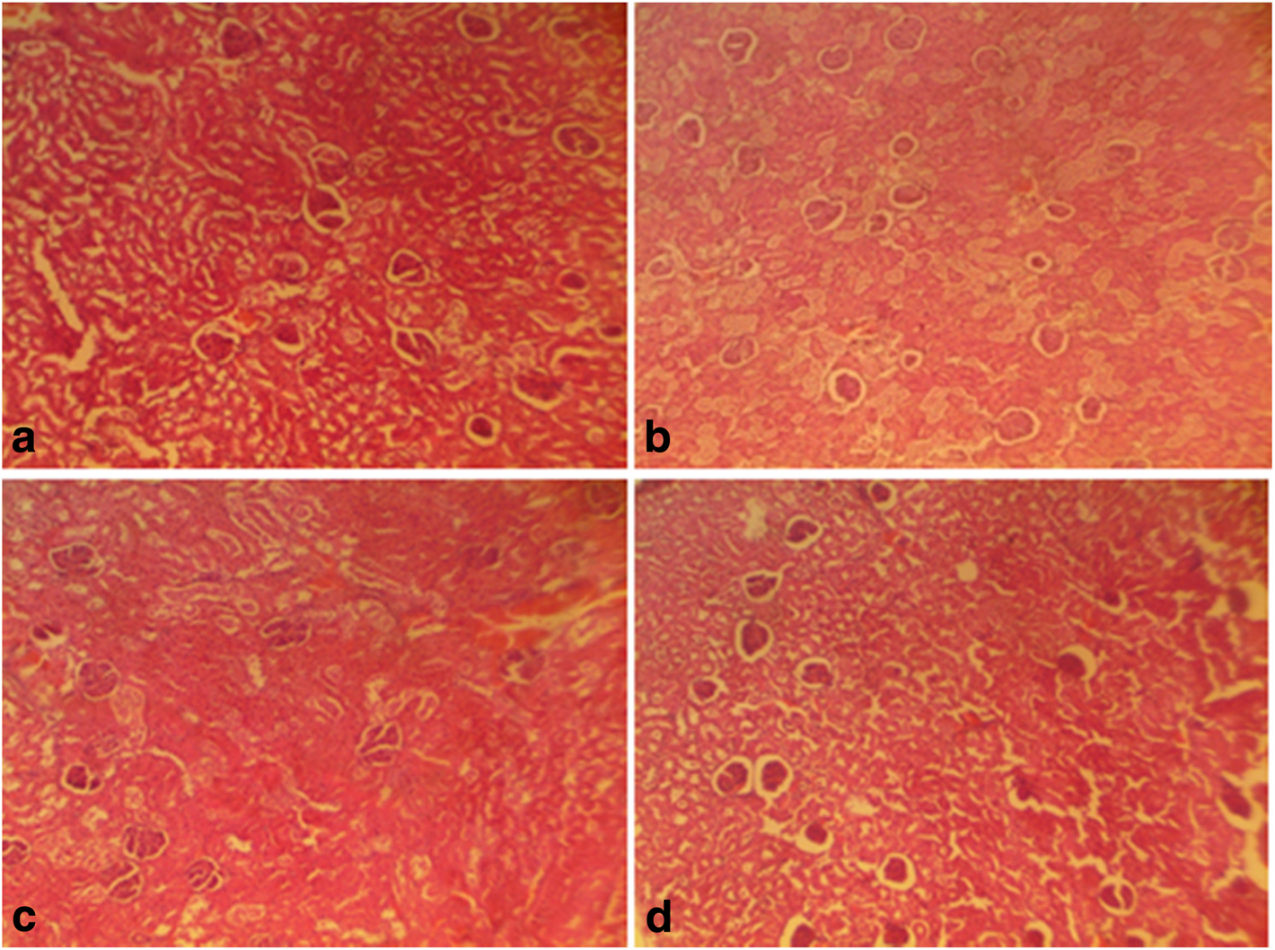
Male rat kidney. Control shows normal kidney (distilled water, 10 ml/kg) (**a**), 1000mg/kg CGV shows normal kidney (**b**), 2000mg/kg CGV shows normal kidney (**c**), 3000mg/kg CGV shows normal kidney (**d**). (H & E, × 400) (CGV: Cellgevity®)

**Fig. 8:**
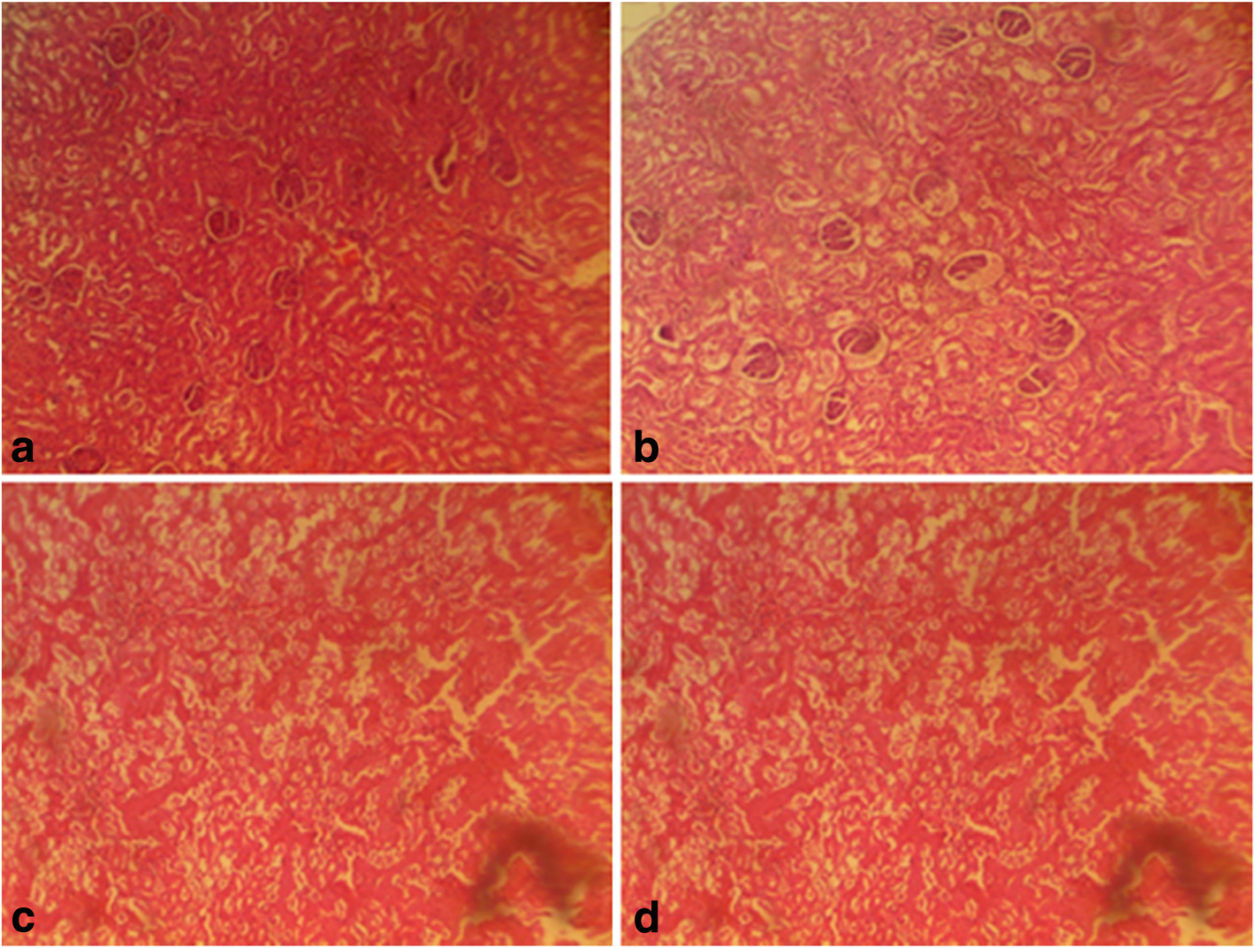
Female rat kidney. Control shows normal kidney (distilled water, 10 ml/kg) (**a**), 1000mg/kg CGV shows normal kidney (**b**), 2000mg/kg CGV shows normal kidney (**c**), 3000mg/kg CGV shows acute tubular necrosis kidney (**d**). (H & E, × 400) (CGV: Cellgevity®)

**Fig. 9:**
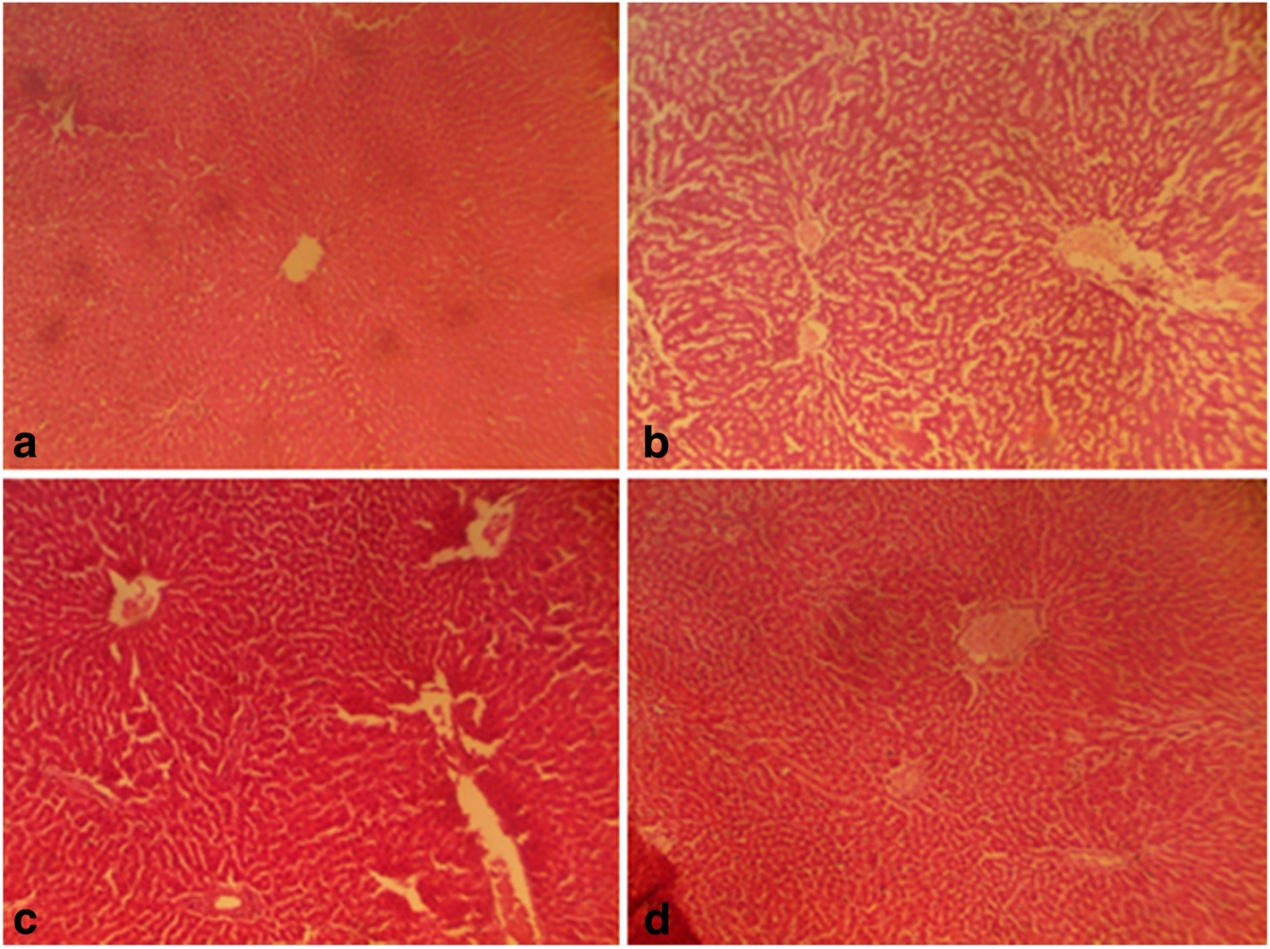
Male rat liver. Control shows normal liver (distilled water, 10 ml/kg) (**a**), 1000mg/kg CGV shows normal liver (**b**), 2000mg/kg CGV shows normal liver (**c**), 3000mg/kg CGV shows normal liver (**d**). (H & E, × 400) (CGV: Cellgevity®)

**Fig. 10:**
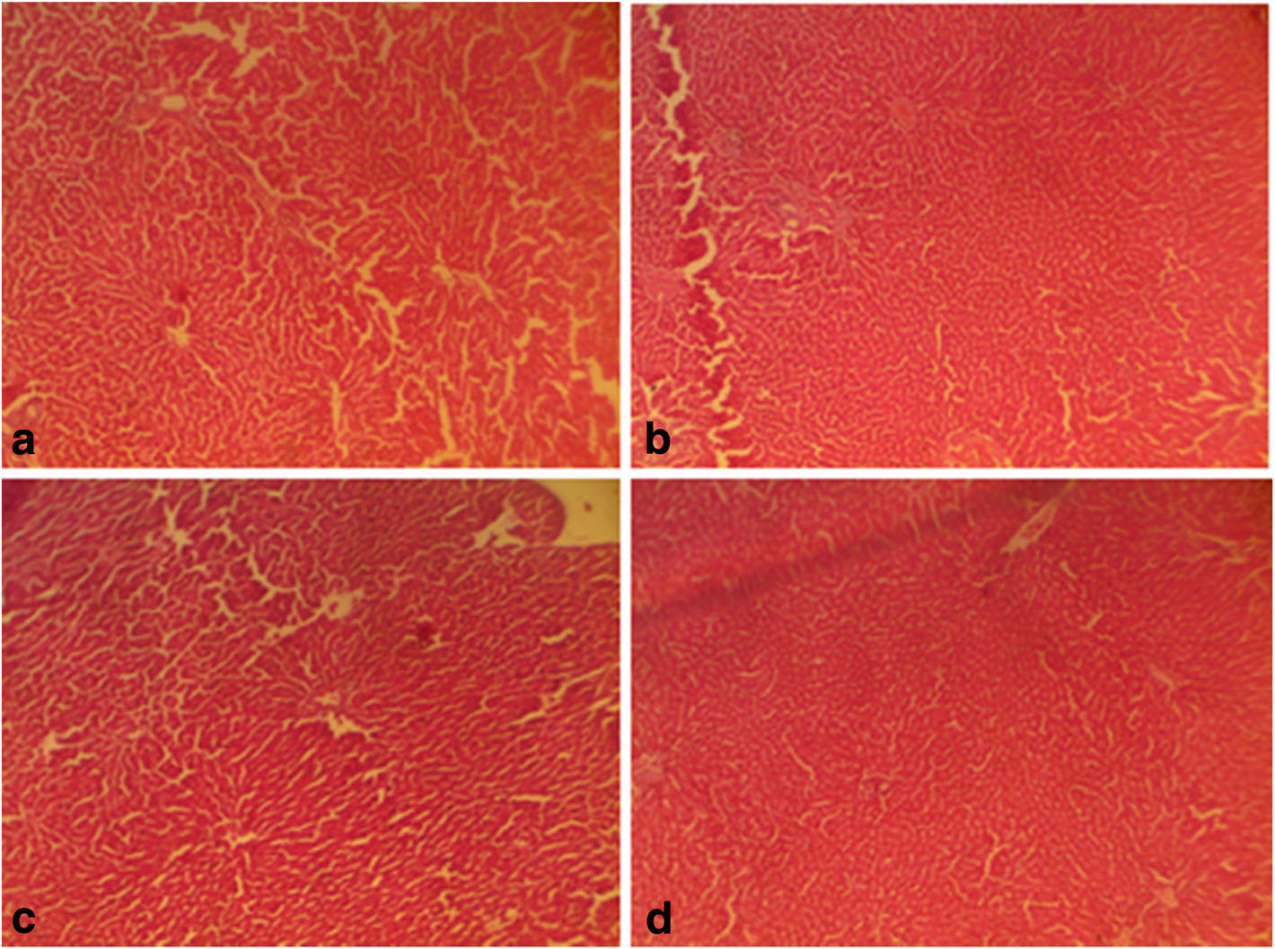
Female rat liver. Control shows normal liver (distilled water, 10 ml/kg) (**a**), 1000mg/kg CGV shows normal liver (**b**), 2000mg/kg CGV shows normal liver (**c**), 3000mg/kg CGV shows normal liver (**d**). (H & E, × 400) (CGV: Cellgevity®)

On the other hand, follicle stimulating hormone remained unaltered given a supra-therapeutic CGV administration. Also, all the supra-therapeutic doses of CGV used in this study did not significantly alter testosterone levels in rats. In female animals, increasing the doses of CGV supra-therapeutically could elevate luteinizing hormone level, whereas such an increase of the same would decrease follicle stimulating hormone level as seen in this study. Also, a similar but dose dependent decrease was obtained in progesterone levels following supra-therapeutic dosing of CGV, whereas, a decrease was observed with estrogen while prolactin remains unperturbed compared with control rats. Although, evidences to support the roles of antioxidants on hormonal regulations are available, however, it may require a chronic administration in order to appreciate the modulatory roles of CGV in this process. The effects of therapeutic and supra-therapeutic doses of CGV on oxidative stress parameters and antioxidant indices in rats were assessed. In rats of both sexes, lipid peroxidation, a biomarker of oxidative stress, measured as malondialdehyde (MDA) was unaltered given the therapeutic doses of CGV when compared with control in this study. Similarly, given the supra-therapeutic doses of CGV, there was no alteration in MDA levels. Pro-oxidants are chemical compounds capable of generating potential toxic oxygen species. Although, a normal cell has an appropriate pro-oxidant–antioxidant balance, however, this balance can be shifted which may not favour the antioxidant system or when levels of antioxidants are diminished [45]. These may result in oxidative stress in which molecular signaling is altered. At this point, since supra-therapeutic doses were ineffective to produce oxidative stress, a very high dose of CGV given for a long period of time may be required. There have been positive correlations between total activities of reduced glutathione, catalase, superoxide dismutase and glutathione peroxidase with total content of MDA in seminal plasma from normozoospermic samples [56]. However, antioxidant enzyme activities were modulated by CGV. For instance, in male rats that were administered supra-therapeutic doses, an increased GSH level was obtained in addition to elevated SOD and CAT activities. Highest doses of CGV reduced GST activity in treated rats. However, at supra-therapeutic doses of CGV administration, MDA levels were elevated. In contrast, SOD and GST activities were slightly reduced both at the therapeutic as well as supra-therapeutic doses of CGV in rats. There are studies to show that numerous cellular processes such as gene expression can influence changes in redox balance where moderate reactive oxygen and reactive nitrogen species production can lead to alterations in cellular and extracellular redox state [57]. This can cause alterations that may signal changes in cell functions. There are chances as obtained in our results that CGV could alter reproductive function in experimental animals. Evidences abound of lowered antioxidant levels in infertile patients suggesting their relationship to male infertility [58]. The difference in body weight following of CGV administration in male and female rats was investigated. CGV supra-therapeutic dosing insignificantly decreased body weight in rats compared with control group. However, at the maximum supra-therapeutic dose used in this study, there was decrease in treated rats of both sexes. A supra-therapeutic 3000 mg/kg dose of CGV increases creatinine level in treated male rats. Also, serum total protein in male animals was reduced (2000 and 3000 mg/kg) in the treated male rats while an elevated low density lipoprotein (3000 mg/kg) was obtained in female animals. However, serum alkaline aminotransferase, total cholesterol triglycerides, albumin, and high density remained unchanged in all animals following therapeutic and supra-therapeutic administrations. CGV administrations seem to modulate biochemical parameters in rats, although, a chronic toxicological administration may be required in order to comments on the possible long term outcome. Histological sections (Figs. 5, 6, 7, 8, 9 and 10) show seminiferous tubules with reduced spermatogenic series with 2000 and 3000 mg/kg respectively (Fig. 5). Also, female rat kidney revealed acute tubular necrosis at highest dose used in this study (Fig. 8). As observed in this study, CGV supra-therapeutic applications could trigger some effects similar to those of pro-oxidants affecting reproductive and antioxidant system. In this current study, we were unable to explore the nueroendocrine effects of supra-therapeutic doses of CGV. More so, direct extrapolation of these findings may be difficult due to physiological differences between rodent and humans.

## Conclusion

Overall, our data further affirmed that CGV is an antioxidant supplement at therapeutic doses. However, the effects on reproductive and biochemical parameters at therapeutic doses are relatively safe during a subchronic administration, although, the pro-oxidant potential of the supra-therapeutic CGV doses was evident as observed is this present study. Hence, it is necessary that its administration be done with caution.

## Abbreviations

CGV: Cellgevity®
ELISA: Enzyme-linked immunosorbent assay
GSH: Reduced glutathione
